# Synthesis and testing of the first azobenzene mannobioside as photoswitchable ligand for the bacterial lectin FimH

**DOI:** 10.3762/bjoc.9.26

**Published:** 2013-02-01

**Authors:** Vijayanand Chandrasekaran, Katharina Kolbe, Femke Beiroth, Thisbe K Lindhorst

**Affiliations:** 1 Christiana Albertina University of Kiel, Otto Diels Institute of Organic Chemistry, Otto-Hahn-Platz 3/4, D-24098 Kiel, Germany, Fax: +49 431 8807410

**Keywords:** azobenzene glycosides, bacterial adhesion, *E*/*Z* photoisomerisation, FimH antagonists, mannobiosides, molecular switches, sweet switches

## Abstract

In order to allow spatial and temporal control of carbohydrate-specific bacterial adhesion, it has become our goal to synthesise azobenzene mannosides as photoswitchable inhibitors of type 1 fimbriae-mediated adhesion of *E. coli*. An azobenzene mannobioside **2** was prepared and its photochromic properties were investigated. The *E*→*Z* isomerisation was found to be highly effective, yielding a long-lived (*Z*)-isomer. Both isomers, *E* and *Z*, show excellent water solubility and were tested as inhibitors of mannoside-specific bacterial adhesion in solution. Their inhibitory potency was found to be equal and almost two orders of magnitude higher than that of the standard inhibitor methyl mannoside. These findings could be rationalised on the basis of computer-aided docking studies. The properties of the new azobenzene mannobioside have qualified this glycoside to be eventually employed on solid support, in order to fabricate photoswitchable adhesive surfaces.

## Introduction

**Figure 1:**
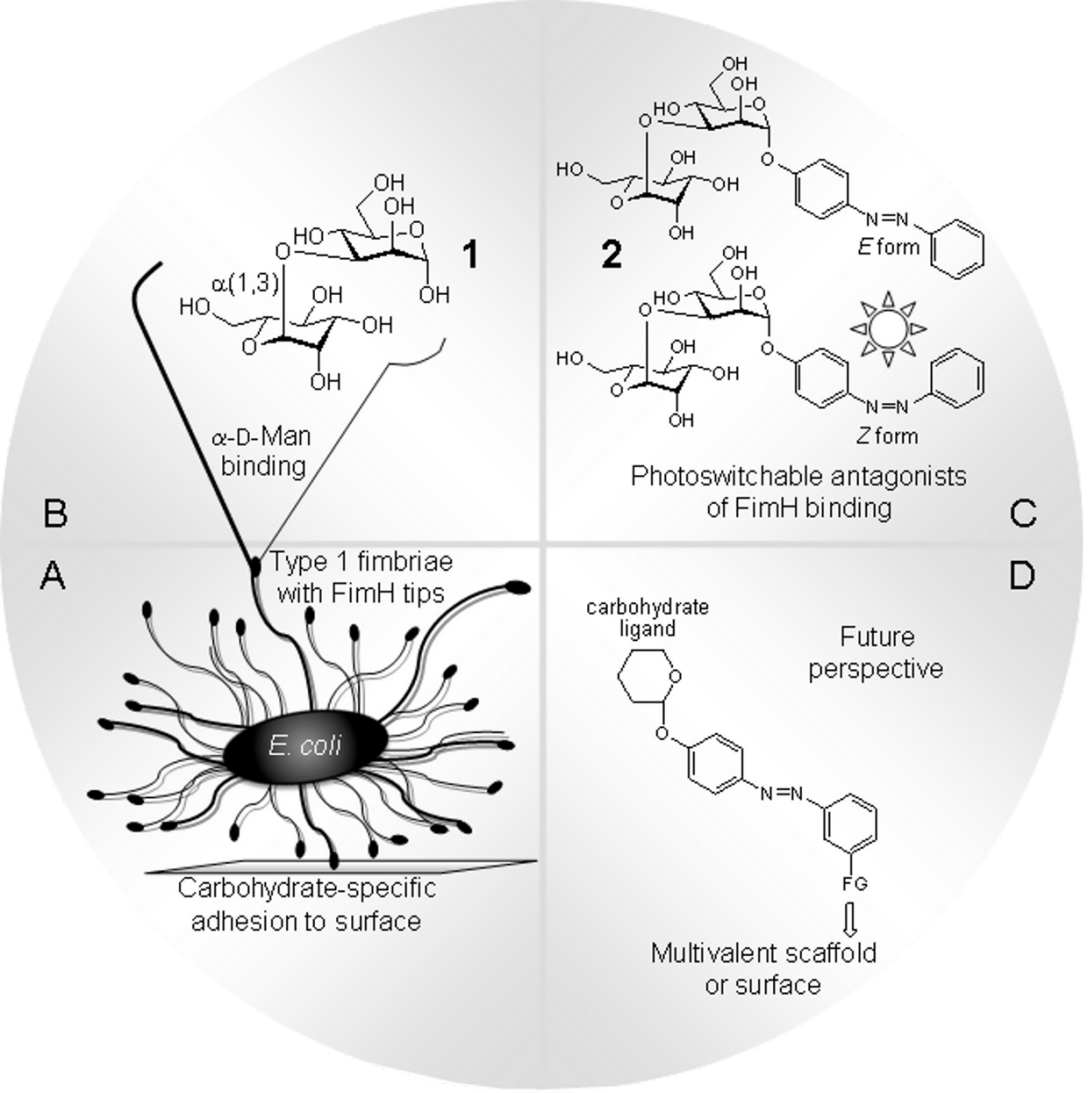
The α-(1→3)-linked mannobioside α-D-Man-(1→3)-D-Man **1** (B) is a potent disaccharide ligand for the bacterial lectin FimH and can thus inhibit type 1 fimbriae-mediated bacterial adhesion to glycosylated surfaces (A). Introduction of an azobenzene aglycone moiety turns glycoside **1** into a putative photoswitchable antagonist **2** of mannose-specific bacterial adhesion, displaying an (*E*)-, as well as a (*Z*)-form (C). In a future perspective azobenzene glycosides such as **2** can be further functionalised to be attached to oligofunctional core molecules or immobilised on surfaces (D).

Adhesion of bacteria to surfaces can be a severe problem both in vivo and in vitro. Hence, inhibition of bacterial adhesion by powerful antagonists is highly desirable, however, ideally on demand, that is, in a specific and spatially as well as temporally resolved way. Often bacterial adhesion depends on the interaction of adhesive organelles called fimbriae. They project from the surface of bacteria and contain lectin domains to attach to certain carbohydrate ligands of a glycosylated surface such as the glycocalyx of eukaryotic target cells (Figure 1A) [1–4]. This offers the possibility to inhibit bacterial adhesion by designed antagonists of the respective carbohydrate-specific bacterial lectins [5]. In order to expand the scope of carbohydrate-based antiadhesives, it has become our goal to make photoswitchable ligands of bacterial lectins to allow blocking of bacterial adhesion in a photocontrolled manner.

One of the best-known fimbriae are the type 1 fimbriae of uropathogenic *E. coli* (UPEC), which comprise the α-D-mannosyl-specific lectin FimH at the tip of the fimbrial shaft. FimH antagonists are currently considered as new therapeutics for the treatment of urinary tract infections [6]. The carbohydrate specificity of FimH has been investigated in great detail [7] and its structure is well-known from several X-ray studies [8–11]. It has turned out that the 1,3-linked mannobioside α-D-Man-(1→3)-D-Man (**1**, Figure 1B) is an ideal disaccharide ligand for FimH [3,12]. All other isomeric mannobiosides do not bind favourably to FimH. Therefore, we have designed the respective azobenzene mannobioside **2** (Figure 1C) in order to make a photoswitchable FimH antagonist available. Photoirradiation of azobenzene glycosides at ~365 nm effects *E*→*Z* isomerisation of the N=N double bond, and thermal relaxation or irradiation at ~450 nm leads to *Z*→*E* back isomerisation [13–14]. In the case that the *E*→*Z* isomerisation process is high-yielding and the lifetime of the (*Z*)-form of the azobenzene glycoside is long enough, it can be employed in bacterial adhesion assays independently from the more stable (*E*)-isomer. Eventually, this type of azobenzene mannobioside can be further functionalised to be attached to various supports such as oligofunctional core molecules [15] or surfaces, to achieve switchable adhesive surfaces in continuation of our work on glycoarrays [16–18] (Figure 1D).

In this account, we describe the synthesis of the azobenzene mannobioside **2** as well as of mannoside **6**, investigation of their photochromic properties, and testing of mannobioside **2** as an inhibitor of type 1 fimbriae-mediated bacterial adhesion. Interpretation of the test results was supported by computer-aided docking studies.

## Results

### Synthesis of azobenzene mannobioside **2**

**Scheme 1:**
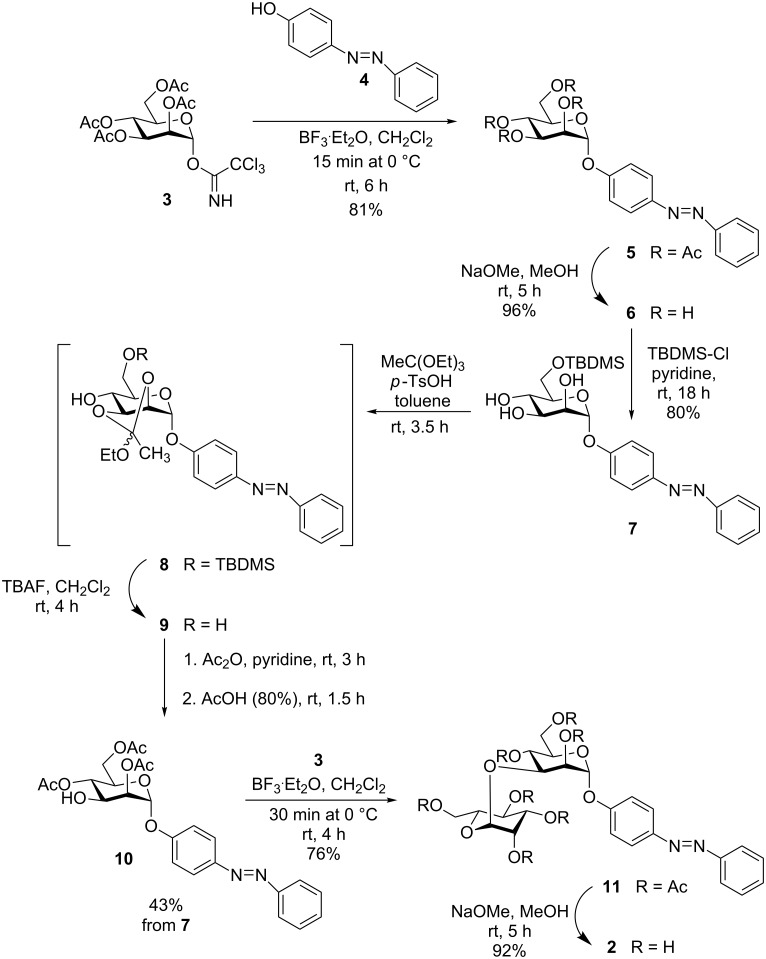
Synthesis of azobenzene mannoside **6** and azobenzene mannobioside **2** by glycosylation.

For the preparation of azobenzene mannobioside **2**, the azobenzene mannoside **6** was prepared first. Thus, mannosylation of the hydroxy-functionalised azobenzene **4** by using the mannosyl trichlororacetimidate **3** [19] led to the respective azobenzene α-mannosides **5** in 81% yield (Scheme 1). Treatment of **5** under Zemplén conditions [20] furnished the deprotected mannoside **6** in a basically quantitative reaction. Then, a standard protecting-group strategy was employed to allow the synthesis of the 3-OH unprotected mannoside **10**, which is a key intermediate serving as the glycosyl acceptor in the following disaccharide synthesis. First, regioselective protection of the primary 6-hydroxy group in **6** was accomplished by using TBDMS chloride in pyridine to yield **7**. Then, triethylorthoacetate was employed to make the orthoester **8**, which, without intermediate purification steps, could be carried on in a sequence of silyl ether-deprotection leading to the intermediate **9**, acetylation of the 4- and 6-hydroxy groups, and then acid-mediated regioselective ring opening of the 2,3-orthoester in the same pot to yield the free 3-OH azobenzene mannoside **10** in an overall yield of 43%. Thus, the required protecting group pattern was obtained in a highly efficient way, based on the regioselective opening of orthoacetates to yield a vicinal arrangement of equatorial OH and axial O-acetyl groups [21–22]. The acetylation pattern was clearly confirmed by ^1^H NMR spectroscopy showing the expected downfield shift for the H-3 signal resonating at 4.32 ppm (H-2: 5.30 ppm, H-4: 5.17 ppm).

Next, glycosylation of the key intermediate **10** by using the mannosyl donor **3** gave the desired mannobioside **11** in 76% yield. Finally, removal of the *O*-acetyl groups according to Zemplén led to the unprotected 1,3-linked target mannobioside α-D-Man-(1→3)-D-Man (**2**).

With the two azobenzene glycosides **6** and **2** at hand, their solubility and photochromic properties were then investigated and compared. Mannoside **6** showed only poor solubility in most organic solvents, except for DMSO. Unfortunately, it was also not soluble in water, or in water/DMSO mixtures, which would allow biological testing. Mannobioside **2**, on the other hand, showed good solubility in polar organic solvents as well as in pure water. Thus, it was amenable to biological testing in aqueous buffer.

*E*→*Z* photoisomerisation of azobenzene mannoside **6** was studied in DMSO, while isomerisation of azobenzene mannobioside **2** was performed in water. Photoirradiation was carried out in the dark at room temperature by employing a 365 nm LED. Photostationary states (PSS) were reached after 10 minutes of irradiation for both compounds. *E*→*Z* isomerisation was observed by both ^1^H NMR and UV–vis spectroscopy. The *E*/*Z* ratios of the ground state (GS) as well as of the photostationary state were determined on the basis of the integration of the anomeric H-1 protons in the ^1^H NMR spectrum. Half-life were determined by UV–vis spectroscopic observation of the thermal *Z*→*E* relaxation process (Supporting Information File 1). The respective data are collected in Table 1.

**Table 1:** Characterisation of the (*E*)- and (*Z*)-isomers of azobenzene glycosides **6** and **2**.

azobenzene glycoside	*E*/*Z*^a^ (GS)	*E*/*Z*^a^ (PSS)	H-1 (ppm) (*E*)*-*isomer	H-1 (ppm) (*Z*)*-*isomer	UV–vis absorption maxima (nm) λ_max_(*E*), λ_max_(*Z*)	half-life, τ_1/2_ (h)

**6**	99:1	3:97	5.54^b^	5.34^b^	347, 440^c^	89
**2**	95:5	4:96	5.65^d^	5.52^d^	339, 429^e^	178.5

^a^according to the integration ratio of H-1(*E*) and H-1(*Z*) in the ^1^H NMR spectrum; ^b^10 mM concentration in DMSO-*d*_6_; ^c^50 µM concentration in DMSO; ^d^8 mM concentration in D_2_O; ^e^65 µM concentration in H_2_O.

Fortunately, the mannobioside **2** is ideally suited for biological testing as it is soluble in water and aqueous buffer, respectively. Photoirradiation of the (*E*)*-*isomer leads to almost quantitative isomerisation, and the life time of the resulting (*Z*)-isomer is long enough to test this isomer independently from the more stable (*E*)-form.

### Biological testing of azobenzene mannobioside **2**

As a test system for mannose-specific bacterial adhesion, fluorescent GFP-transfected *E. coli* bacteria (pPKL1162) [23] were employed and tested on a mannan-coated polystyrene microtiter plate surface. In this setup the amount of bacterial adhesion correlates with fluorescence intensity and can be quantified by using a standard microtiter plate reader. For inhibition of bacterial adhesion, two sets of serially diluted solutions of **2** were prepared to inhibit adhesion of fluorescing *E. coli* to the mannan surface. In one case, a stock solution of (*E*)-**2** was serially diluted, in the second case, this stock solution of (*E*)-**2** was irradiated for 15 minutes to obtain the pure (*Z*)**-2** isomer for subsequent serial dilution. The effect of both isomers as inhibitors of mannose-specific bacterial adhesion was then measured in a concentration-dependent way. From the testing results sigmoidal inhibition curves were obtained (Supporting Information File 1) from which IC_50_ values for every individual inhibitor were deduced. The IC_50_ value reflects the concentration at which a compound inhibits 50% of bacterial adhesion to a mannan-coated surface. The determined IC_50_ values were referenced to the inhibitory potency of methyl α-D-mannoside (MeMan) and *p*-nitrophenyl α-D-mannoside (*p*NPMan), respectively, each tested on the same plate. Thus, relative inhibitory potencies (RIP values) were obtained, which allow comparison of inhibitory potencies of different inhibitors, even when they were not tested in the same experiment. The testing results collected in Table 2 show that the inhibitory power of mannobioside **2** is roughly the same, regardless of whether its (*E*)- or (*Z*)-form was employed. Inspection of their relative inhibitory potencies reveals that both isomers of **2** are equally potent inhibitors of type 1 fimbriae-mediated bacterial adhesion, similar to the power of the well-known mannoside *p*NPMan.

**Table 2:** Inhibition of adhesion of *E. coli* to a mannan-coated surface. The inhibitory potencies of (*E*)- and (*Z*)-**2** are compared to the standard inhibitors MeMan and *p*NPMan. ^a^

	MeMan	*p*NPMan	(*E*)-**2**	(*Z*)-**2**

IC_50_ ± SD (mM)	5.205 ± 0.416		0.064 ± 0.018	0.073 ± 0.001

		0.073 ± 0.003	0.078 ± 0.006	0.084 ± 0.002

RIP (MeMan) ± SD	IP ≡ 1		81 ± 25	71 ± 1
RIP (*p*NPMan) ± SD		IP ≡ 1	0.94 ± 0.07	0.87 ± 0.02

^a^Average values from duplicate results; SD: standard deviation (from one assay); RIP: relative inhibitory potency referenced to either MeMan or *p*NPMan, each tested on the same microtiter plate.

In order to support the interpretation of the obtained test results, binding of (*E*)-**2** and (*Z*)-**2** to the bacterial lectin FimH was investigated by computer-aided docking studies to get an idea of their interactions with the carbohydrate-recognition domain (CRD) of the lectin.

### Docking of azobenzene mannobioside **2** into the carbohydrate binding site of FimH

To visualise complexation of the (*E*)- and (*Z*)-isomers of azobenzene mannobioside **2** within the CRD of FimH FlexX [24–26], flexible docking and consensus scoring [27–28], as implemented in Sybyl 6.9 [29], was employed. Docking was based on two different X-ray structures of FimH. They differ in the conformation of the so-called tyrosine gate at the entrance of the CRD, formed by the side chains of Y48 and Y137. One structure is crystallised in an “open-gate” conformation [9], another in the “closed-gate” conformation [10]. Affinity of any FimH ligand is improved when it exerts favourable interactions with the tyrosine gate of FimH. Thus, this substructure is an important feature of the rim of the carbohydrate binding site of this lectin.

Before minimisation of the ligands, the bond angle of the N=N double bond of the azobenzene moiety was manually set as 180° for (*E*)-**2** and as 90° for (*Z*)-**2** [30]. Then docking was performed holding the FimH CRD fixed whereas the ligands were allowed to change their conformations under the influence of the force field. A FlexX scoring value has been attributed to each of the 30 obtained conformations (Table 3). This value correlates with the binding affinity of the ligand for the FimH CRD, more negative values suggesting higher binding affinity than less negative ones.

**Table 3:** FlexX scoring values for the (*E*)- and the (*Z*)-isomer of **2** based on two different crystal structures in comparison to MeMan and *p*NPMan.

Ligand	“open-gate” structure [9]	“closed-gate” structure [10]

MeMan	−22.5	−23.3
*p*NPMan	−24.9	−27.4
(*E*)-**2**	−28.8	−20.4
(*Z*)-**2**	−28.7	−21.6

**Figure 2:**
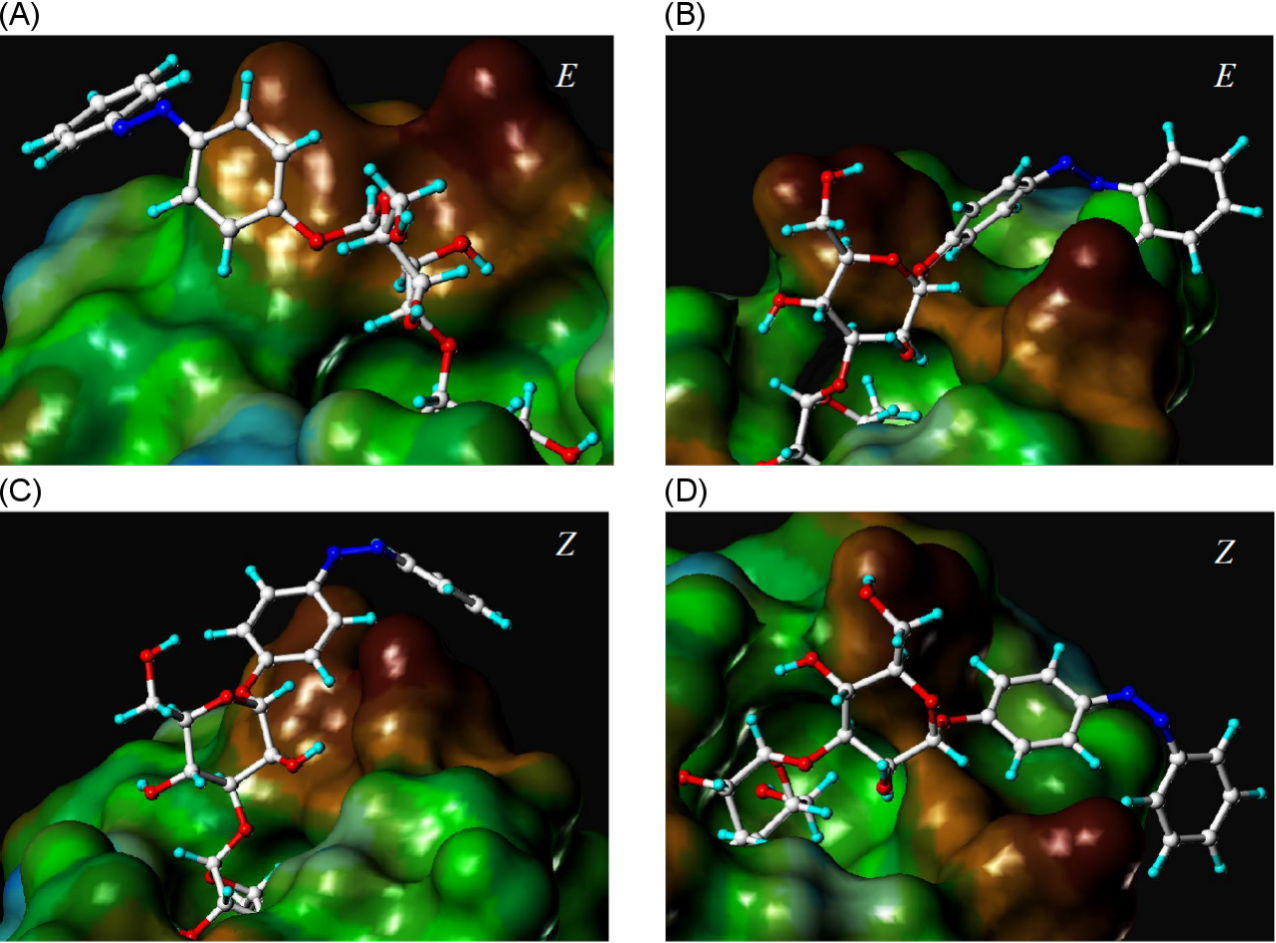
Connolly [32–33] descriptions of the FimH CRD with the docked azobenzene mannobioside **2**. Top: (*E*)-isomer (A, closed-gate; B, open-gate conformation). Bottom: (*Z*)-isomer (C, closed-gate; D, open-gate conformation).

Docking gave very similar results for both isomers of mannobioside **2**, (*E*)-**2** and (*Z*)-**2**. Scoring values based on the open-gate structure of FimH are almost equal (−28.8 and −28.7), and also the scoring values obtained with the closed-gate structure do not differ significantly (−20.4 and −21.6). Interestingly, the predictions for *p*NPMan and also MeMan are the opposite, suggesting better binding to the closed-gate conformation of FimH, as described earlier [31]. Representative snapshots as depicted in Figure 2 show that both isomers have the terminal mannoside complexed within the CRD of the lectin, as expected, and furthermore, that in both cases the azobenzene moiety exerts effective interactions with the tyrosine gate involving both benzene rings.

Regardless of whether the (*E*)- or the (*Z*)-form of **2** is complexed with FimH, favourable π–π interactions can be formed between the azobenzene moiety and the tyrosine gate at the entrance of the CRD, though in different ways. The only difference that is seen is that, apparently, the interactions of mannobioside **2** with the open-gate conformation of FimH are advantageous over those with the closed-gate form. From the bioassay in solution phase it can certainly not be decided, which conformation the bacterial lectin adopts to interact with compound **2**; however, our test results confirm that both isomers of the azobenzene mannobioside **2** have the same power as inhibitors of FimH-mediated bacterial adhesion.

## Discussion

The azobenzene mannobioside **2** was selected as a photoswitchable inhibitor of the bacterial lectin FimH based on earlier findings about the inhibitory potency of several mannobiosides [34–35]. Its synthesis was straightforward and high-yielding. It has very convenient photochromic properties as the *E*→*Z* isomerisation is almost quantitative and the resulting (*Z*)-isomer is especially long-lived. Both isomers are very well water-soluble and could be independently tested as inhibitors of mannose-specific bacterial adhesion and showed an equal and high inhibitory potency in the range of the well-known high-affinity inhibitor *p*-nitrophenyl α-D-mannoside (*p*NPMan). This result can be rationalised by computer docking, showing that regardless of the configuration of the N=N double bond of the azobenzene moiety in **2**, favourable interactions can be formed with the tyrosine gate of the FimH CRD. While the terminal mannoside portion is complexed in the carbohydrate binding site, the first mannoside does not add significantly to the affinity and this is in accordance with other studies on the complexation of oligosaccharides by FimH [11]. However, this mannose ring acts as a spacer moiety, sticking out straight from the CRD and placing the azobenzene portion in an orientation that allows flexible interactions with the tyrosine gate at the entrance of the carbohydrate binding site of the lectin.

Apparently, the affinity of **2** to the open-gate form of FimH is higher than to the closed-gate conformation, a finding that differs from many other docked FimH ligands. Here, the higher affinity for the open-gate FimH can be explained by strong π–π stacking of the first aromatic ring of the azobenzene unit with the tyrosine gate.

As both isomers of **2** interact equally well with FimH, they can’t be used to switch type 1 fimbriae-mediated bacterial adhesion in solution. On the other hand, the obtained results support the idea to immobilise the azobenzene mannobioside on a solid support to photocontrol the adhesive properties of the resulting surface. In this approach the azobenzene N=N double bond can be used as a hinge region to bend down the terminal mannose moiety of the compound, which is critical for specific bacterial adhesion. Thus, upon *E*→*Z* isomerisation, the ligand will no longer be available for the interaction with the FimH-terminated type 1 fimbriae that mediate adhesion. In this approach, the second mannose moiety of the mannobioside is important both to mediate hydrophilicity and to intensify the steric effect that photoswitching has on the exposition of the terminal mannoside.

## Conclusion

The azobenzene mannosides presented herein resemble a structure quite similar to biaryl mannosides, which have been introduced lately and shown to be of medical relevance as FimH antagonists [6]. Thus, our novel “sweet switches” [15] appear to be highly promising FimH ligands, with the additional feature of a photoswitchable moiety. The biomedicinal potential of azobenzene glycosides seems even higher when their favourable physiological properties are considered, such as low toxicity [36] and receptor specificity of the azobenzene aglycon [37]. It will be our next goal to employ derivatives of azobenzene mannobioside **2** for immobilisation to test the photoswitching of adhesion on surfaces.

## Experimental

### Materials and general methods

*p*-Hydroxyazobenzene was purchased from Sigma Aldrich and used without further purification. Moisture-sensitive reactions were carried out under nitrogen in dry glassware. Thin-layer chromatography was performed on silica-gel plates (GF 254, Merck). Detection was effected by UV and/or charring with 10% sulfuric acid in EtOH followed by heat treatment at ~180 °C. Flash chromatography was performed on silica gel 60 (Merck, 230–400 mesh, particle size 0.040–0.063 mm) by using distilled solvents. Optical rotations were measured with a Perkin-Elmer 241 polarimeter (sodium D-line: 589 nm, length of cell: 1 dm) in the solvents indicated. ^1^H and ^13^C NMR spectra were recorded on Bruker DRX-500 and AV-600 spectrometers at 300 K. Chemical shifts are reported relative to internal tetramethylsilane (δ = 0.00 ppm) or D_2_O (δ = 4.76 ppm). Full assignment of the peaks was achieved with the aid of 2D NMR techniques (^1^H/^1^H COSY and ^1^H/^13^C HSQC). IR spectra were measured with a Perkin Elmer FT-IR Paragon 1000 (ATR) spectrometer. ESI mass spectra were recorded on an Esquire-LC instrument from Bruker Daltonics. MALDI-TOF mass spectra were recorded on a Bruker Biflex III instrument with 19 kV acceleration voltage, and 2,5-dihydroxybenzoic acid (DHB) was used as the matrix. UV–vis absorption spectra were performed on Perkin-Elmer Lambda-241 or Varian Cary-5000 at a temperature of 18 ± 1 °C. Photoirradiaton was carried out by using a LED (emitting 365 nm light) from the Nichia Corporation (NC4U133A) with a FWHM of 9 nm and an optical power output (*P*_o_) ~ 1 W.

For NMR assignments the following numbering was used:


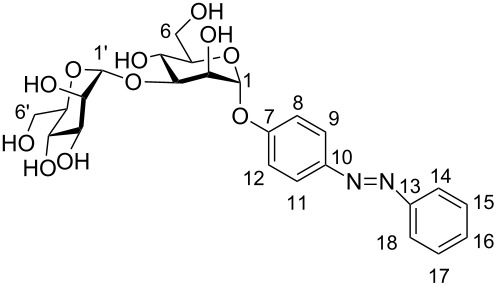


**(*E*)-*p*-(Phenylazo)phenyl 2,3,4,6-tetra-*O*-acetyl-α-D-mannopyranoside (5).** To a solution of the mannosyl donor **3** (5.00 g, 10.2 mmol) and *p*-hydroxyazobenzene (**4**, 2.01 g, 10.2 mmol) in dry CH_2_Cl_2_ (100 mL) BF_3_·etherate (1.88 mL, 15.2 mmol) was added at 0 °C under N_2_ atmosphere, and the reaction mixture was stirred at this temperature for 15 min. Then, stirring was continued at rt for about 6 h, and then the reaction was quenched by the addition of satd. aq. NaHCO_3_ solution (50 mL). The phases were separated, the aqueous phase was extracted with CH_2_Cl_2_ (2 × 150 mL), and the combined organic phases were dried over MgSO_4_. This was filtered, and the filtrate was concentrated under reduced pressure. Purification of the crude product by column chromatography (cyclohexane/ethyl acetate, 3:1) gave the title glycoside **5** as an orange crystalline solid (4.33 g, 8.19 mmol, 81%). Mp 53–55 °C; *R*_f_ 0.35 (cyclohexane/ethyl acetate 2:1); [α]^20^_D_ +0.86 (*c* 0.9, DMSO); ^1^H NMR (500 MHz, CDCl_3_) δ 7.92 (d, *J* = 9.0 Hz, 2H, H-9, H-11), 7.89 (d, *J* = 8.5 Hz, 2H, H-14, H-18), 7.53–7.45 (m, 3H, H-15, H-16, H-17), 7.23 (d, *J* = 9.0 Hz, 2H, H-8, H-12), 5.62 (d, *J*_1,2_ = 1.8 Hz, 1H, H-1), 5.58 (dd, *J*_2,3_ = 3.6 Hz, *J*_3,4_ = 10.1, 1H, H-3), 5.49 (dd, *J*_1,2_ = 1.8 Hz, *J*_2,3_ = 3.6 Hz, 1H, H-2), 5.39 (dd~t, *J*_3,4_ = *J*_4,5_ = 10.0 Hz, 1H, H-4), 4.30 (dd, *J*_5,6a_ = 5.4 Hz, *J*_6a,6b_ = 12.0 Hz, 1H, H-6a), 4.14–4.07 (m, 2H, H-5, H-6b), 2.21, 2.06, 2.05, 2.03 (each s, each 3H, 4 OAc); ^13^C NMR (125 MHz, CDCl_3_) δ 170.5, 169.9, 169.9, 169.7 (4 C=O), 157.6 (C-7), 152.6 (C-13), 148.4 (C-10), 130.8 (C-16), 128.9 (C-15, C-17), 124.6 (C-9, C-11), 123.1 (C-14, 18), 116.8 (C-8, C-12), 95.7 (C-1), 69.4 (C-5), 69.3 (C-2), 68.8 (C-3), 65.9 (C-4), 62.1 (C-6), 20.9, 20.7, 20.7, 20.6 (4 COCH_3_); IR (ATR) 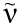: 2929, 1743, 1598, 1496, 1366, 1209, 1029 cm^−1^; ESIMS (*m*/*z*): [M + Na]^+^ calcd for C_26_H_28_N_2_O_10_, 551.5; found, 551.1.

**(*E*)-*p*-(Phenylazo)phenyl α-D-mannopyranoside (6).** To a solution of the acetyl-protected glycoside **5** (600 mg, 1.14 mmol) in dry MeOH (6 mL), a catalytic amount of solid NaOMe was added under N_2_ atmosphere, and the reaction mixture was stirred for 5 h at rt. Then it was neutralized with Amberlite IR 120 ion-exchange resin and filtered. The filtrate was evaporated under reduced pressure to yield the deprotected mannoside **6** as a pale yellow solid (393 mg, 1.09 mmol, 96%). Mp 183–185 °C; *R*_f_ 0.34 (ethyl acetate/MeOH 4:1); [α]^20^_D_ +1.40 (*c* 1.0, DMSO); ^1^H NMR (500 MHz, DMSO-*d*_6_) δ 7.86 (d, *J* = 8.7 Hz, 2H, H-9, H-11), 7.82 (d, *J* = 7.7 Hz, 2H, H-14, H-18), 7.53–7.45 (m, 3H, H-15, H-16, H-17), 7.26 (d, *J* = 8.7 Hz, 2H, H-8, H-12), 5.53 (bs, 1H, H-1), 3.89 (dd~bs, 1H, H-2), 3.73 (dd, *J*_3,4_ = 9.2 Hz, *J*_3,2_ = 3.0 Hz, 1H, H-3), 3.58–3.47 (m, 3H, H-6a, H-4, H-6b), 3.38 (m_c_, 1H, H-5); ^13^C NMR (125 MHz, DMSO-*d*_6_) δ 158.9 (C-7), 155.5 (C-13), 151.9 (C-10), 130.9 (C-16), 129.4 (C-15, C-17), 124.3 (C-9, C-11), 122.3 (C-14, C-18), 117.1 (C-8, C-12), 98.7 (C-1), 75.2 (C-5), 70.6 (C-3), 69.9 (C-2), 66.6 (C-4), 60.9 (C-6); UV, λ_max_: 347 nm; ε = 25907 ± 529 L × mol^−1^ × cm^−1^; IR (ATR) 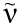: 3337, 2920, 1599, 1584, 1496, 1227 cm^−1^; MALDI-TOFMS (*m*/*z*): [M + H]^+^ calcd for 361.36; found, 361.21; anal. calcd for C_18_H_20_N_2_O_6_: C, 59.99; H, 5.59; N, 7.77; found: C, 61.07; H, 5.80; N, 8.07.

**NMR spectroscopic data for (*Z*)-6. **^1^H NMR (500 MHz, DMSO-*d*_6_) δ 7.32 (t, *J* = 7.8 Hz, 2H, H-15, H-17), 7.18 (t, *J* = 7.4 Hz, 1H, H-16), 6.97 (d, *J* = 8.9 Hz, 2H, H-9, H-11), 6.82 (dd, *J* = 8.2 Hz, *J* = 6.7 Hz, 4H, H-8, H-12, H-14, H-18), 5.32 (d, *J*_1,2_ = 1.6 Hz, 1H, H-1), 3.77 (dd, *J*_2,3_ = 3.1 Hz, *J*_1,2_ = 1.9 Hz, 1H, H-2), 3.62 (dd, *J*_3,4_ = 9.3 Hz, *J*_3,2_ = 3.3 Hz, 1H, H-3), 3.52 (dd, *J*_5,6a_ = 2.1 Hz, *J*_6a,6b_= 11.8 Hz, 1H, H-6a), 3.48–3.36 (m, 2H, H-4, H-6b), 3.31 (ddd, *J*_4_*,*_5_ = 9.4 Hz, *J*_5,6a_ = 5.8 Hz, *J*_5,6b_ = 2.1 Hz, 1H, H-5); ^13^C NMR (125 MHz, DMSO-*d*_6_) δ 155.4 (C-7), 153.8 (C-13), 147.3 (C-10), 129.1 (C-15, C-17), 127.0 (C-16), 122.5 (C-8, C-12), 119.4 (C-14, C-18), 116.7 (C-9, C-11), 98.7 (C-1), 74.8 (C-5), 70.3 (C-3), 69.7 (C-2), 66.3 (C-4), 60.7 (C-6); UV, λ_max_: 440 nm, ε = 2635 ± 76 L × mol^−1^ × cm^−1^.

**(*E*)-*p*-(Phenylazo)phenyl 6-*O*-*tert*-butyldimethylsilyl-α-D-mannopyranoside (7).** To a solution of the azobenzene mannoside **6** (3.00 g, 8.33 mmol) in pyridine (30.0 mL) *tert*-butyldimethylchlorosilane (1.38 g, 9.17 mmol) was added and the reaction mixture was stirred at rt for 18 h, after which TLC showed complete consumption of the starting material. The reaction was quenched with MeOH (2.0 mL) and further diluted with ethyl acetate (150 mL). Then it was washed with satd. aq. NaHCO_3_ solution (30 mL) and the aqueous phase extracted with ethyl acetate (2 × 50 mL). The combined organic phases were dried over MgSO_4_ and filtered, and the filtrate concentrated under reduced pressure to obtain the crude product. Purification by flash column chromatography (CH_2_Cl_2_/MeOH 3:7) gave the title compound as a dark orange solid (3.16 g, 6.66 mmol, 80%). Mp 74 °C; *R*_f_ 0.59 (CH_2_Cl_2_/MeOH 7:1); [α]^20^_D_ +73 (*c* 0.97, MeOH); ^1^H NMR (500 MHz, MeOH-*d*_4_) δ 7.94–7.88 (m, 4H, H-9, H-11, H-14, H-18), 7.57–7.49 (m, 3H, H-15, H-16, H-17), 7.30 (d, *J* = 9.0 Hz, 2H, H-8, H-12), 5.62 (d, *J*_1,2_ = 1.7 Hz, 1H, H-1), 4.08 (dd, *J*_1,2_ = 1.8 Hz, *J*_2,3_ = 3.4 Hz, 1H, H-2), 3.98 (dd, *J*_5,6a_ = 1.8 Hz, *J*_6a,6b_ = 11.2 Hz, 1H, H-6a), 3.94 (dd, *J*_2,3_ = 3.5 Hz, *J*_3,4_ = 9.1 Hz, 1H, H-3), 3.82 (dd, *J*_5,6b_ = 6.5 Hz, *J*_6a,6b_ = 11.3 Hz, 1H, H-6b), 3.71 (t, *J* = 9.4 Hz, 1H, H-4), 3.65 (m_c_, 1H, H-5), 0.83 (s, 9H, *tert-*butyl), 0.04, 0.05 (each s, each 3H, 2 Si-CH_3_) ppm; ^13^C NMR (125 MHz, MeOH-*d*_4_) δ 160.4 (C-7), 154.1 (C-13), 149.18 (C-10), 131.8 (C-16), 130.2 (C-15), 125.5 (C-17), 123.6 (C-9), 118.2 (C-11), 100.0 (C-1), 76.2 (C-5), 72.3 (C-2), 71.7 (C-3), 68.6 (C-4), 64.4 (C-6), 26.4 (C(CH_3_)_3_), 19.1 (C(CH_3_)_3_), −5.13 (2 Si-CH_3_) ppm; IR (ATR) 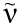: 3337, 2928, 1599, 1498, 1229, 1006, 685 cm^−1^; ESIMS (*m*/*z*): [M + Na]^+^ calcd for C_24_H_34_N_2_O_6_Si, 497.1; found, 497.2;.

**(*E*)-*p*-(Phenylazo)phenyl 6-*O*-*tert*-butyldimethylsilyl-2,3-*O*-(ethylorthoacetyl)-α-D-mannopyranoside (8).** To a solution of mannoside **7** (500 mg, 1.05 mmol) in toluene (8.0 mL), triethylorthoacetate (773 µL, 4.22 mmol) and a catalytic amount of *p*-toluenesulfonic acid were added at rt, and the reaction mixture was stirred for 3.5 h, after which TLC showed complete consumption of the starting material. Then, it was neutralised with triethylamine (100 µL), and the solution was diluted with water (10 mL). It was extracted with toluene (2 × 20 mL), and the extract was concentrated under reduced pressure to get crude **8** (600 mg) as a red viscous syrup, which was used in the next reaction step without further purification.

**(*E*)-*p*-(Phenylazo)phenyl 2,3-*O*-(ethylorthoacetyl)-α-D-mannopyranoside (9).** The crude intermediate **8** (600 mg) was dissolved in CH_2_Cl_2_ (6.0 mL), tetrabutylammonium fluoride (1 M solution in THF, 1.68 mL) was added, and the reaction mixture was stirred at rt for 4 h, after which TLC showed complete consumption of the starting material. Then, it was concentrated under reduced pressure to obtain crude **9** as a dark red viscous syrup (594 mg), which was used in the next reaction step without further purification.

**(*E*)-*p*-(Phenylazo)phenyl 2,4,6-tri-*O*-acetyl-α-D-mannopyranoside (10).** The crude orthoester-protected mannoside **9** (594 mg) was dissolved in pyridine (2.5 mL), and acetic anhydride (1.26 mL) was added for O-acetylation. The reaction mixture was stirred at rt for 3 h. Then, pyridine was removed under reduced pressure, and the residue was dissolved in ethyl acetate (20 mL) and washed with satd. aq. NaHCO_3_ solution (10 mL). The aqueous phase was extracted with ethyl acetate (2 × 25 mL), the combined organic phases were dried over Na_2_SO_4_ and filtered, and the filtrate concentrated was under reduced pressure to obtain a syrupy intermediate. It was dissolved in 80% acetic acid (2.5 mL), and the mixture was stirred at rt for 1.5 h to effect regioselective cleavage of the orthoester. Then, ethyl acetate (50 mL) was added and the organic layer was washed with water (5 mL) and dried over MgSO_4_. It was filtered, and the filtrate was evaporated to obtain the crude product, which purified by column chromatography (cyclohexane/ethyl acetate 2:1) to yield the free 3-OH title mannoside **10** as a bright orange solid (220 mg, 0.453 mmol, 43% over three steps). Mp 144–146 °C; *R*_f_ 0.21 (cyclohexane/ethyl acetate); [α]^20^_D_ +70 (*c* 0.96, CH_2_Cl_2_); ^1^H NMR (500 MHz, CDCl_3_) δ 7.92 (d, *J* = 8.9 Hz, 2H, H-9, H-11), 7.89 (d, *J* = 7.9 Hz, 2H, H-14, H-18), 7.53–7.44 (m, 3H, H-15, H-16, H-17), 7.19 (d, *J* = 8.9 Hz, 2H, H-8, H-12), 5.69 (d, *J*_1,2_ = 1.4 Hz, 1H, H-1), 5.30 (dd, *J*_1,2_ = 1.7 Hz, *J*_2,3_ = 3.8 Hz, 1H, H-2), 5.17 (t, *J* = 10.0 Hz, 1H, H-4), 4.32 (m, 2H, H-3, H-6a), 4.11 (dd, *J*_5,6b_ = 2.2 Hz, *J*_6a,6b_ = 12.4 Hz, 1H, H-6b), 4.05 (m_c_, 1H, H-5), 2.23, 2.15, 2.03 (each s, each 3H, 3 OAc), 1.62 (bs, OH) ppm; ^13^C NMR (150 MHz, CDCl_3_) δ 171.3, 170.6, 170.4 (3 COCH_3_), 157.7 (C-7), 152.6 (C-13), 148.3 (C-10), 130.8 (C-16), 129.1 (C-15, C-17), 124.6 (C-9, C-11), 122.7 (C-14, C-18), 116.7 (C-8, C-12), 95.42 (C-1), 71.99 (C-2), 69.14 (C-5), 69.04 (C-4), 68.47 (C-3), 62.15 (C-6), 20.97, 20.91, 20.69 (3 COCH_3_) ppm; IR (ATR) 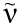: 3453, 2961, 1737, 1228, 1023, 798 cm^−1^; ESIMS (*m*/*z*): [M + H]+ calcd for C_24_H_26_N_2_O_9_, 509.1; found, 509.2.

**(*E*)-*p*-(Phenylazo)phenyl 3-*O*-(2,3,4,6-tetra-*O*-acetyl-α-D-mannopyranosyl)-2,4,6-tri-*O*-acetyl-α-D-mannopyranoside (11).** The 3-OH unprotected mannoside **10** (50 mg, 103 µmol) and the mannosyl donor **3** (101 mg, 206 µmol) were dissolved in dry CH_2_Cl_2_ (10 mL), and the mixture was cooled to −10 °C under N_2_ atmosphere. To this ice-cooled solution BF_3_·etherate (13 µL, 108 µmol) was added and the mixture was stirred at 0 °C for about 30 min. Then, the reaction mixture was allowed to warm to rt and stirred for another 4 h. The reaction mixture was then quenched by the addition of a catalytic amount of solid NaHCO_3_ and concentrated under reduced pressure to obtain the crude product as a dark reddish-brown syrup. Purification by column chromatography (CH_2_Cl_2_/ethyl acetate 8:2) gave the acetyl-protected mannobioside **11** as a pale yellow solid (64 mg, 78 µmol, 76%). Mp 84–85 °C; *R*_f_ 0.57 (CH_2_Cl_2_/ethyl acetate 8:2); [α]^20^_D_ +103 (*c* 0.86, CH_2_Cl_2_); ^1^H NMR (500 MHz, CDCl_3_) δ 7.92 (d, *J* = 9.0 Hz, 2H, H-9, H-11), 7.89 (d, *J* = 7.1 Hz, 2H, H-14, H-18), 7.46–7.38 (m, 3H, H-15, H-16, H-17), 7.18 (d, *J* = 9.0 Hz, 2H, H-8, H-12), 5.65 (d, *J*_1,2_ = 1.7 Hz, 1H, H-1), 5.46 (dd, *J*_1,2_ = 1.8 Hz, *J*_2,3_ = 3.5 Hz, 1H, H-2), 5.40 (t, *J* = 10.1 Hz, 1H, H-4′), 5.30 (m_c_, 1H, H-3′), 5.26 (m_c_, 1H, H-4), 5.09 (d, *J*_1,2_ = 1.7 Hz 1H, H-1′), 5.06 (dd, *J*_1,2_ = 1.9 Hz, *J*_2,3_ = 2.9 Hz, 1H, H-2′), 4.41 (dd, *J*_2,3_ = 3.5 Hz, *J*_3,4_ = 9.9 Hz, 1H, H-3), 4.30 (dd, *J*_5,6b_ = 6.3 Hz, *J*_6a,6b_ = 12.7 Hz, 1H, H-6a), 4.24 (dd, *J*_5′,6b′_ = 5.8 Hz, *J*_6a′,6b′_ = 12.3 Hz, 1H, H-6a′), 4.14–4.10 (m, H-5′, H-6b), 4.08 (dd, *J*_5,6a_ = 2.4 Hz, *J*_6a,6b_ = 12.3 Hz, 1H, H-6b), 3.99 (ddd, *J*_4,5_ = 10.2 Hz, *J*_5,6a_ = 2.3 Hz, *J*_6a,6b_ = 5.8 Hz, 1H, H-5), 2.20, 2.09, 2.08, 2.03, 2.00, 1.97, 1.94 (each s, each 3H, 7 OAc) ppm; ^13^C NMR (125 MHz, CDCl_3_) δ 170.6, 170.5, 170.4, 170.0, 169.9, 169.8, 169.6 (7 COCH_3_), 157.4 (C-7), 152.6 (C-13), 148.4 (C-10), 130.8 (C-16), 129.1 (C-15, C-17), 124.6 (C-9, C-11), 122.71 (C-14, C-18) 116.7 (C-8, C-12), 99.1 (C-1′), 95.6 (C-1), 74.8 (C-3), 70.8 (C-2), 69.9 (C-2′), 69.9, 69.6 (C-5, C-5′), 68.3 (C-4), 67.4 (C-4′), 65.9 (C-3′), 62.5, 62.7 (C-6, C-6′), 20.9, 20.8, 20.7, 20.7, 20.7, 20.6, 20.6 (7 COCH_3_) ppm; IR (ATR) 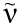: 1743, 1213, 1032, 838 cm^−1^; ESIMS (*m*/*z*): [M + Na]^+^ calcd for C_38_H_44_N_2_O_18_, 839.3; found, 839.2.

**(*E*)-*p*-(Phenylazo)phenyl 3-*O*-(α-D-mannopyranosyl)-α-D-mannopyranoside (2).** The acetyl-protected disaccharide **11** (50 mg, 61.2 µmol) was dissolved in dry MeOH (2 mL) and a catalytic amount of solid NaOMe was added under N_2_ atmosphere. The reaction mixture was stirred for 5 h at rt, and then it was neutralized with Amberlite IR 120 ion-exchange resin. It was then filtered and thoroughly washed with MeOH (2 × 20 mL), and the filtrate was evaporated to obtain the crude product, which after purification by flash column chromatography (CH_2_Cl_2_/methanol 9:1) gave the final mannobioside **2** as a pale yellow solid (29.3 mg, 56.1 µmol, 92%). Mp 107–109 °C; *R*_f_ 0.08 (CH_2_Cl_2_/MeOH 9:1); [α]^20^_D_ +18.4 (*c* 0.48, MeOH); ^1^H NMR (500 MHz, D_2_O) δ 7.81 (d, *J* = 8.2 Hz, 2H, H-9, H-11), 7.75 (d, *J* = 7.4 Hz, 2H, H-14, H-18), 7.53–7.49 (m, 3H, H-15, H-16, H-17), 7.24 (d, *J* = 8.3 Hz, 2H, H-8, H-12), 5.65 (s, 1H, H-1), 5.16 (s, 1H, H-1′), 4.29 (m_c_, 1H, H-2), 4.14 (dd, *J*_2,3_ = 3.1 Hz, *J*_3,4_ = 10.3 Hz, 1H, H-3′), 4.06 (m_c_, 1H, H-2′), 3.89–3.81 (m, 3H, H-3, H-4, H-4′), 3.79–3.62 (m, 6H, H-6a, H-6b, H-5, H-5′, H-6a′, H-6b′) ppm; ^13^C NMR (125 MHz, D_2_O) δ 158.2 (C-7), 151.3 (C-13), 148.2 (C-10), 131.4 (C-16), 129.6 (C-15, C-17), 124.4 (C-9, C-11), 122.2 (C-14, C-18), 117.3 (C-8, C-12), 102.4 (C-1′), 97.8 (C-1), 77.9 (C-3′), 73.8 (C-5), 73.5 (C-3), 70.5 (C-4′), 70.1 (C-2′), 69.5 (C-2), 66.9 (C-5′), 65.9 (C-4), 61.1 (C-6), 60.6 (C-6′) ppm; IR (ATR) 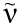: 3318, 2927, 1599, 1231, 1007, 685 cm^−1^; MALDI-TOFMS (*m*/*z*): [M + Na]^+^ calcd for C_24_H_30_N_2_O_11_, 545.18; found, 545.17; UV, λ_max_: 339 nm, ε = 14776 ± 729 L × mol^−1^ × cm^−1^; anal. calcd for C_24_H_30_N_2_O_11_ × 1.1 H_2_O: C, 52.11; H, 6.09; N, 5.07; found: C, 52.04; H, 5.79; N, 5.06.

**NMR-spectroscopic data for (*Z*)-2.** ^1^H NMR (500 MHz, D_2_O) δ 7.32 (t, *J* = 7.1 Hz, 2H, H-15, H-17), 7.24 (t, 1H, H-16), 7.00 (dd, *J* = 1.9 Hz, *J* = 8.9 Hz, 2H, H-9, H-11), 6.95 (dd, *J* = 1.9 Hz, *J* = 8.9 Hz, 2H, H-8, H-12), 6.91 (dd, *J* = 1.3 Hz, *J* = 7.8 Hz, 2H, H-14, H-18), 5.52 (s, 1H, H-1), 5.12 (s, 1H, H-1′), 4.22 (m_c_, 1H, H-2), 4.07 (m_c_, 1H, H-3′), 4.03 (dd, *J*_1,2_ = 1.7 Hz, *J*_2,3_ = 3.2 Hz, 1H, H-2′), 3.85–3.82 (m, 2H, H-3, H-4), 3.79–3.59 (m, 7H, H-4′, H-6a, H-6b, H-5, H-5′, H-6a′, H-6b′) ppm; ^13^C NMR (125 MHz, D_2_O) δ 155.3 (C-7), 153.5 (C-13), 146.9 (C-10), 129.2 (C-15, C-17), 128.2 (C-16), 123.4 (C-8, C-12), 120.3 (C-14, C-18), 116.9 (C-9, C-11), 102.4 (C-1′), 97.8 (C-1), 77.8 (C-3′), 73.7 (C-5), 73.4 (C-3), 70.4 (C-4′), 70.1 (C-2′), 69.4 (C-2), 66.8 (C-5′), 65.9 (C-4), 61.0 (C-6), 60.6 (C-6′) ppm; UV, λ_max_: 429 nm, ε = 1699 ± 68 L × mol^−1^ × cm^−1^.

## Supporting Information

File 1 Photoisomerization studies, UV–vis spectra, NMR spectra, bioassay and docking results.
