# Supplementary material for: Synthesis and testing of the first azobenzene mannobioside as photoswitchable ligand for the bacterial lectin FimH
Source: Beilstein J Org Chem. 2013 Feb 1;9:223–33. doi: 10.3762/bjoc.9.26 (PMC3566865; doi:10.3762/bjoc.9.26)
Supplement: File 1 — Photoisomerization studies, UV–vis spectra, NMR spectra, bioassay and docking results. [file Beilstein_J_Org_Chem-09-223-s001.pdf]

# Supporting Information

## for

# Synthesis and testing of the first azobenzene mannobioside as photoswitchable ligand for the bacterial lectin FimH

Vijayanand Chandrasekaran, Katharina Kolbe, Femke Beiroth and Thisbe K. Lindhorst*

Address: Christiana Albertina University of Kiel, Otto Diels Institute of Organic Chemistry, Otto-Hahn-Platz 3/4, D-24098 Kiel, Germany, Fax: +49 431 8807410

E-mail: Thisbe K. Lindhorst - tklind@oc.uni-kiel.de

*Corresponding author

## Photoisomerization studies, UV–vis spectra, NMR spectra, bioassay and docking results

## Table of contents

1. **Data of photoisomerisation** ...... S2
   UV–vis spectra of thermal (*Z*)-**6**→(*E*)-**6** relaxation ...... S2
   UV–vis spectra of thermal (*Z*)-**2**→(*E*)-**2** relaxation ...... S3
   $^{1}$H and $^{13}$C NMR spectra of (*E*)-**6** and (*Z*)-**6** ...... S4
   $^{1}$H and $^{13}$C NMR spectra of (*E*)-**2** and (*Z*)-**2** ...... S5
2. **Bioassays** ...... S6
3. **Docking studies** ...... S8
4. **References** ...... S12

## 1. Data of photoisomerization

Photoirradiation experiments were carried out at room temperature by using 365 nm LED lights (Product Number: NC4U133A) from Nichia Corporation (NJSE107) with power dissipation 1.4 W and luminous flux 44 [lm]. Photoisomerization experiments with compound **6** were performed in DMSO and with compound **2** in water. Upon irradiaton, the photostationary state (PSS) was within 10 min for both compounds, as observed by UV–vis spectroscopy, The *E*→*Z* isomerisation process is reflected by a decrease of the π–π* transition and an increase in the n–π* transition band.

The kinetics of the *Z*→*E* relaxation process was determined by UV–vis spectroscopy in the dark. For determination of the rate constants, a graph of $\ln(A_\infty - A_t)$ was plotted as a function of time; where $A_\infty$ is the absorbance of the π–π* transition at infinitive time and $A_t$ is the absorbance at time *t* after the relaxation process was started. The negative slope *k* of the linear plot is the rate constant of the *Z*→*E* relaxation process. The half life $\tau_{1/2}$ as $\tau_{1/2} = \ln 2/k$.

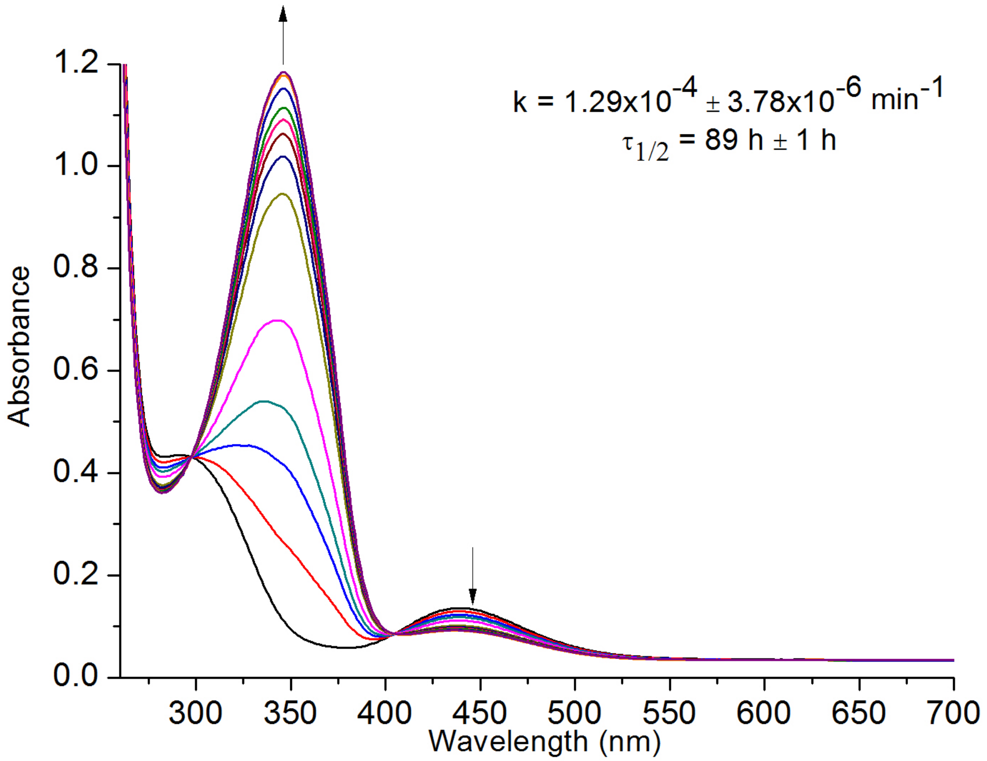


**Figure S1:** UV–vis spectra of thermal *Z*→*E* relaxation of mannoside **6** in DMSO (50 µM) at 18 ± 1 °C.

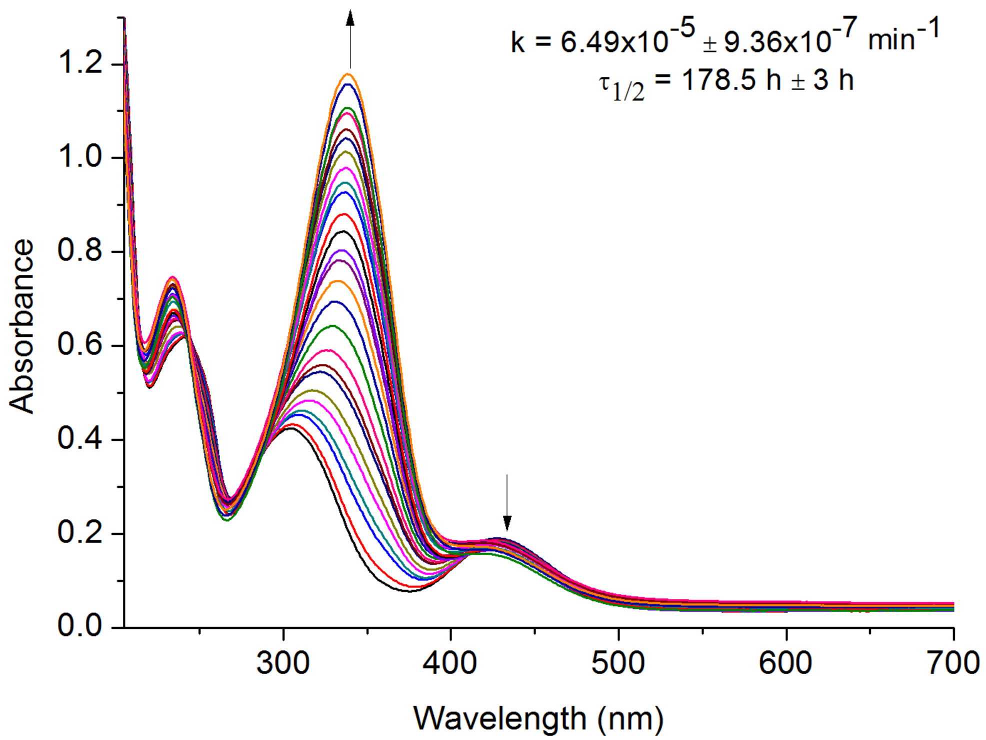


**Figure S2:** UV–vis spectra of thermal $Z \rightarrow E$ relaxation of the mannobioside **2** in water (65 µM) at 18 ± 1 °C.

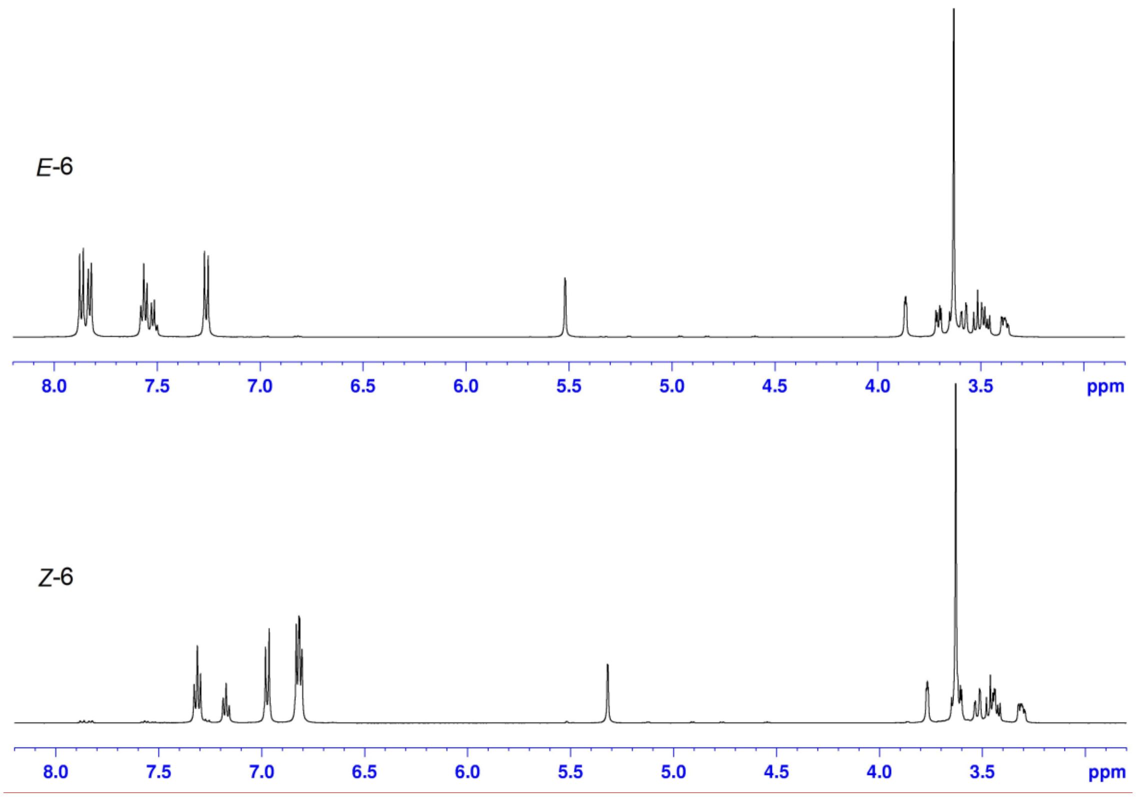


**Figure S3:** $^{1}$H NMR spectra of (*E*)-**6** and (*Z*)-**6** in DMSO-$d_6$ (500 MHz).

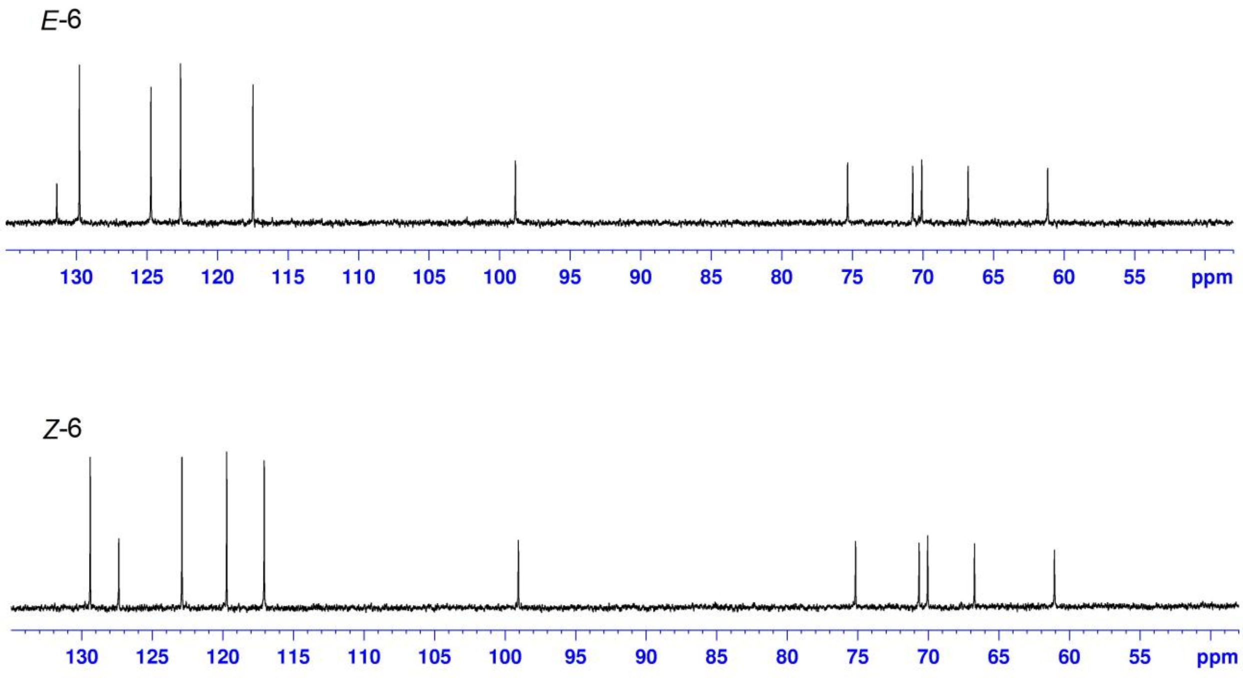


**Figure S4:** $^{13}$C NMR spectra of (*E*)-**6** and (*Z*)-**6** in DMSO-$d_6$ (125 MHz).

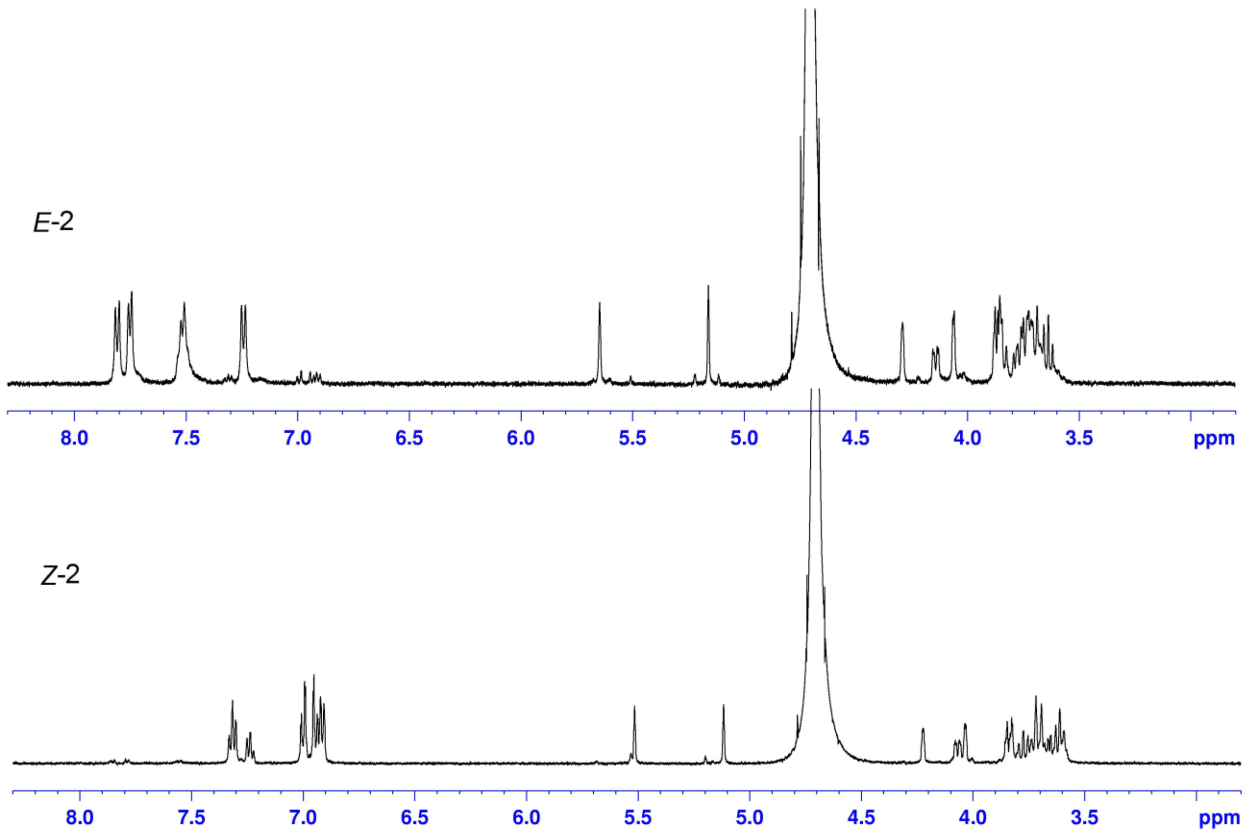


**Figure S5:** $^{1}$H NMR spectra of (*E*)-**2** and (*Z*)-**2** in $D_2O$ (500 MHz).

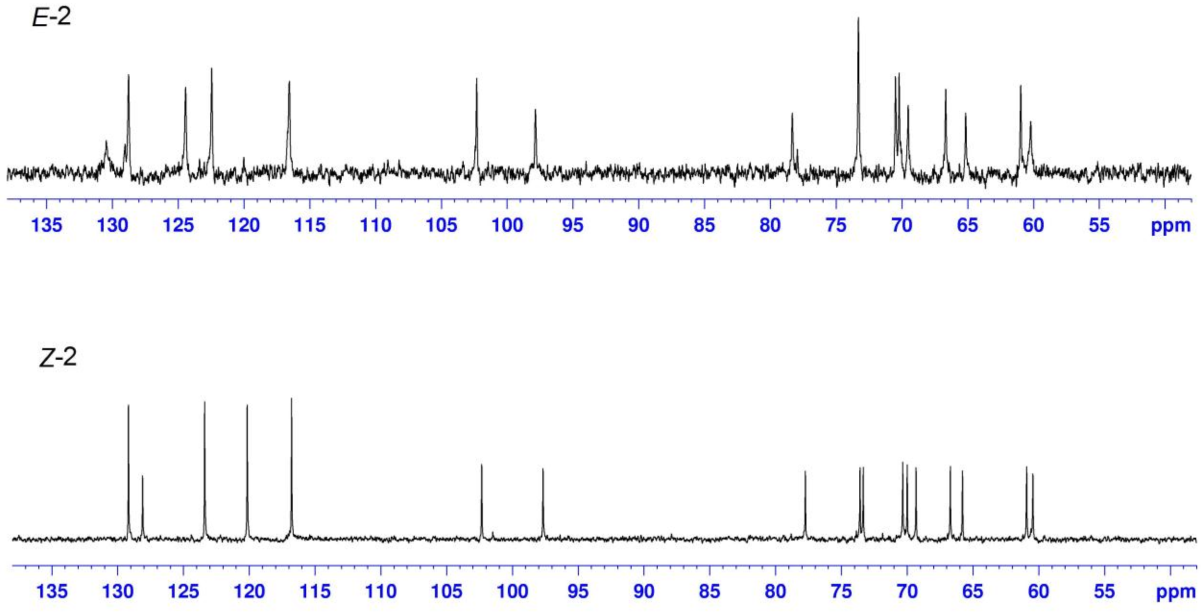


**Figure S6:** $^{13}$C NMR spectra of (*E*)-**2** and (*Z*)-**2** in $D_2O$ (125 MHz).

## 2. Bioassays

**Media and buffer solutions**: Carbonate buffer solution (pH 9.6): sodium carbonate (1.59 g) and sodium hydrogen carbonate (2.52 g) were dissolved in double-distilled water (1.00 L). PBS buffer solution (pH 7.2): sodium chloride (8.00 g), potassium chloride (200 mg), sodium hydrogen phosphate dihydrate (1.44 g) and potassium dihydrogenphosphate (200 mg) were dissolved in double-distilled water (1.00 L). PBST buffer solution (pH 7.2): PBS buffer + 0.05% v/v Tween® 20. LB medium: tryptone (10.0 g), sodium chloride (10.0 g) and yeast extract (5.00 g) were dissolved in distilled, deionised water (1.00 L); after autoclaving, ampicillin (100 mg) and chloramphenicol (50.0 mg) were added. pH values were adjusted by using 0.1 M HCl or 0.1 M NaOH.

**Cultivation of bacteria**: *E. coli* bacteria (strain pPKL1162) [1] were grown on LB medium overnight at 37 °C in a sterilized tube. After centrifugation and washing with PBS buffer (2 × 2.00 mL) the bacteria pellet was suspended to a concentration of 2.00 mg/mL in PBS buffer.

**GFP assay**: The published assay [2] was adapted and modified as follows: Black (nunc Maxisorp) plates were treated with a solution of mannan from *Saccharomyces cerevisiae* (1.2 mg/mL in carbonate buffer, pH 9.5; 100 µL/well) and allowed to dry at 37 °C overnight. The plates were washed with PBST (3 × 150 µL/well). Before use the wells were blocked with BSA (5% in PBS, 120 µL/well) for 2 h at 37 °C and then washed with PBST (3 × 150 µL/well). Serial dilutions of the examined inhibitor mannobioside **2** ((*E*)- or (*Z*)-configured, respectively) were prepared in the plates (50 µL/well). The bacteria suspension (2 mg bacteria/mL, 50 µL/well) was added and the plates were agitated (80 rpm) and incubated for 1 h at 37 °C. After washing with PBS (2 × 150 µL) the wells were filled with PBS (100 µL/well) and the fluorescence intensity (485 nm/535 nm) was determined.

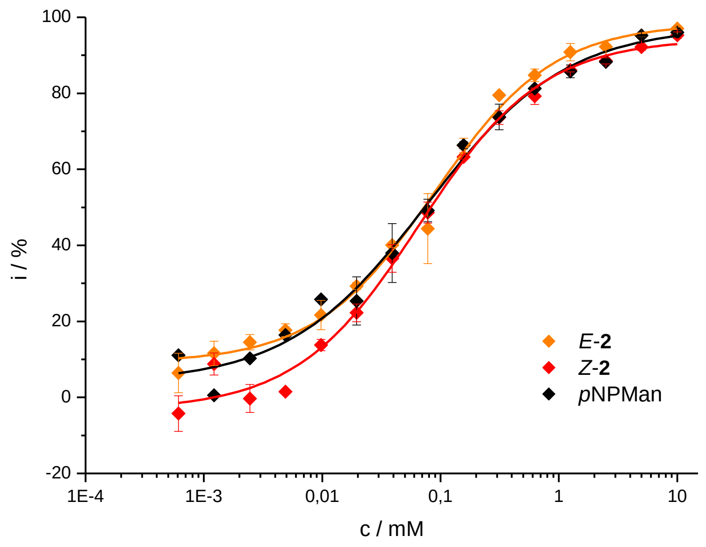


**Figure S7:** Inhibition curves obtained in inhibition of adhesion of *E. coli* to a mannan-coated surface. The isomers (*E*)- and (*Z*)-**2** were tested on one microtiter plate together with *p*NPMan. The sigmoidal concentration–response curves were fitted by nonlinear regression.

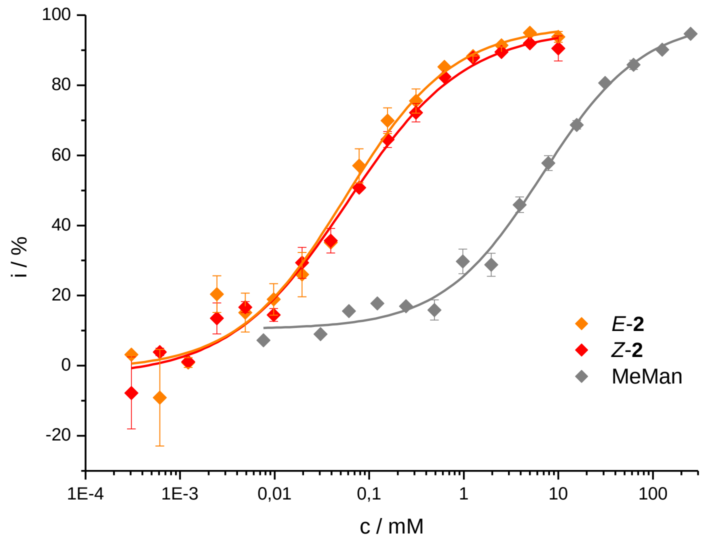


**Figure S8:** Inhibition curves obtained in inhibition of adhesion of *E. coli* to a mannan-coated surface. The isomers (*E*)- and (*Z*)-**2** were tested on one microtiter plate together with MeMan. The sigmoidal concentration–response curves were fitted by nonlinear regression.

## 3. Docking studies

Computer-aided docking was performed by using FlexX flexible docking and consensus scoring, as implemented in Sybyl 6.9 [3] as described previously [4]. Docking was based on two published X-ray structures of the bacterial lectin FimH [5] (1KLF (open-gate) and 1UWF (closed-gate structure)). The torsion angle of the azobenzene moiety was manually set up to 180° for the (*E*)- and to 90° for the (*Z*)-isomer and then the structures were minimized by using the Tripos force field and Gasteiger–Hückel charges. Thirty conformations and FlexX scoring values were obtained for each ligand and listed in Tables S1–S4.

**Table S1:** Scoring values for docking of *E*-configured azobenzene mannobioside **2** into the closed-gate structure of FimH.

| No. | Total Score | Match-Score | Lipo-Score | Ambig-Score | Clash-Score | Rot-Score | RMS-Value | Simil. Index | #Match | Avg. Volume | Max. Volume | Frag. No. |
|---|---|---|---|---|---|---|---|---|---|---|---|---|
| 1 | -20.362 | -25.378 | -12.299 | -8.232 | 1.947 | 18.200 | 0.000 | -1.000 | 9 | 0.045 | 0.666 | 1 |
| 2 | -19.480 | -28.399 | -10.811 | -8.923 | 5.053 | 18.200 | 5.165 | -1.000 | 10 | 0.193 | 2.362 | 1 |
| 3 | -18.806 | -23.699 | -12.232 | -8.767 | 2.292 | 18.200 | 0.816 | -1.000 | 8 | 0.049 | 0.840 | 1 |
| 4 | -18.641 | -25.472 | -11.712 | -8.529 | 3.472 | 18.200 | 0.563 | -1.000 | 10 | 0.127 | 1.369 | 1 |
| 5 | -18.416 | -24.813 | -11.531 | -8.295 | 2.622 | 18.200 | 0.825 | -1.000 | 9 | 0.053 | 0.710 | 1 |
| 6 | -18.401 | -25.638 | -10.708 | -7.968 | 2.313 | 18.200 | 1.574 | -1.000 | 9 | 0.072 | 1.215 | 1 |
| 7 | -18.063 | -23.870 | -11.586 | -9.123 | 2.917 | 18.200 | 0.904 | -1.000 | 9 | 0.099 | 1.916 | 1 |
| 8 | -18.047 | -28.399 | -9.790 | -8.512 | 5.053 | 18.200 | 4.341 | -1.000 | 10 | 0.193 | 2.362 | 1 |
| 9 | -17.876 | -28.399 | -9.822 | -8.309 | 5.053 | 18.200 | 4.908 | -1.000 | 10 | 0.193 | 2.362 | 1 |
| 10 | -17.872 | -28.399 | -9.817 | -8.309 | 5.053 | 18.200 | 4.922 | -1.000 | 10 | 0.193 | 2.362 | 1 |
| 11 | -17.859 | -28.399 | -9.810 | -8.306 | 5.056 | 18.200 | 4.911 | -1.000 | 10 | 0.193 | 2.362 | 1 |
| 12 | -17.832 | -27.788 | -10.267 | -8.836 | 5.459 | 18.200 | 3.758 | -1.000 | 10 | 0.237 | 2.362 | 1 |
| 13 | -17.800 | -28.928 | -7.061 | -8.124 | 2.714 | 18.200 | 3.619 | -1.000 | 14 | 0.096 | 1.580 | 1 |
| 14 | -17.744 | -27.788 | -9.883 | -9.132 | 5.459 | 18.200 | 3.807 | -1.000 | 10 | 0.237 | 2.362 | 1 |
| 15 | -17.676 | -28.399 | -9.579 | -8.351 | 5.053 | 18.200 | 4.356 | -1.000 | 10 | 0.193 | 2.362 | 1 |
| 16 | -17.563 | -29.810 | -5.148 | -7.249 | 1.044 | 18.200 | 7.255 | -1.000 | 10 | 0.067 | 1.818 | 1 |
| 17 | -17.563 | -29.810 | -5.148 | -7.249 | 1.044 | 18.200 | 7.015 | -1.000 | 10 | 0.067 | 1.818 | 1 |
| 18 | -17.563 | -29.810 | -5.148 | -7.249 | 1.044 | 18.200 | 5.507 | -1.000 | 10 | 0.067 | 1.818 | 1 |
| 19 | -17.563 | -29.810 | -5.148 | -7.249 | 1.044 | 18.200 | 6.413 | -1.000 | 10 | 0.067 | 1.818 | 1 |
| 20 | -17.563 | -29.810 | -5.148 | -7.249 | 1.044 | 18.200 | 7.271 | -1.000 | 10 | 0.067 | 1.818 | 1 |
| 21 | -17.563 | -29.810 | -5.148 | -7.249 | 1.044 | 18.200 | 7.057 | -1.000 | 10 | 0.067 | 1.818 | 1 |
| 22 | -17.563 | -29.810 | -5.148 | -7.249 | 1.044 | 18.200 | 5.813 | -1.000 | 10 | 0.067 | 1.818 | 1 |
| 23 | -17.563 | -29.810 | -5.148 | -7.249 | 1.044 | 18.200 | 5.763 | -1.000 | 10 | 0.067 | 1.818 | 1 |
| 24 | -17.563 | -29.810 | -5.148 | -7.249 | 1.044 | 18.200 | 7.050 | -1.000 | 10 | 0.067 | 1.818 | 1 |
| 25 | -17.563 | -29.810 | -5.148 | -7.249 | 1.044 | 18.200 | 6.432 | -1.000 | 10 | 0.067 | 1.818 | 1 |
| 26 | -17.563 | -29.810 | -5.148 | -7.249 | 1.044 | 18.200 | 7.000 | -1.000 | 10 | 0.067 | 1.818 | 1 |
| 27 | -17.563 | -29.810 | -5.148 | -7.249 | 1.044 | 18.200 | 6.404 | -1.000 | 10 | 0.067 | 1.818 | 1 |
| 28 | -17.563 | -29.810 | -5.148 | -7.249 | 1.044 | 18.200 | 5.785 | -1.000 | 10 | 0.067 | 1.818 | 1 |
| 29 | -17.563 | -29.810 | -5.148 | -7.249 | 1.044 | 18.200 | 5.823 | -1.000 | 10 | 0.067 | 1.818 | 1 |
| 30 | -17.563 | -29.810 | -5.148 | -7.249 | 1.044 | 18.200 | 7.052 | -1.000 | 10 | 0.067 | 1.818 | 1 |

**Table S2:** Scoring values for docking of *E*-configured azobenzene mannobioside **2** into the open-gate structure of FimH.

| No. | Total Score | Match-Score | Lipo-Score | Ambig-Score | Clash-Score | Rot-Score | RMS-Value | Simil. Index | #Match | Avg. Volume | Max. Volume | Frag. No. |
|---|---|---|---|---|---|---|---|---|---|---|---|---|
| 1 | -28.809 | -34.291 | -10.848 | -9.010 | 1.740 | 18.200 | 0.000 | -1.000 | 16 | 0.040 | 0.577 | 1 |
| 2 | -28.340 | -34.839 | -11.028 | -9.298 | 3.224 | 18.200 | 0.553 | -1.000 | 17 | 0.112 | 1.245 | 1 |
| 3 | -28.331 | -34.037 | -10.560 | -9.118 | 1.784 | 18.200 | 0.312 | -1.000 | 17 | 0.042 | 0.600 | 1 |
| 4 | -28.006 | -34.637 | -11.348 | -8.848 | 3.227 | 18.200 | 0.451 | -1.000 | 16 | 0.115 | 1.271 | 1 |
| 5 | -27.439 | -34.726 | -12.221 | -9.279 | 5.186 | 18.200 | 0.753 | -1.000 | 19 | 0.188 | 1.635 | 1 |
| 6 | -27.439 | -34.726 | -12.221 | -9.279 | 5.186 | 18.200 | 0.754 | -1.000 | 19 | 0.188 | 1.635 | 1 |
| 7 | -27.310 | -34.110 | -12.764 | -9.021 | 4.986 | 18.200 | 0.973 | -1.000 | 19 | 0.195 | 1.803 | 1 |
| 8 | -27.310 | -34.110 | -12.764 | -9.021 | 4.986 | 18.200 | 0.977 | -1.000 | 19 | 0.195 | 1.803 | 1 |
| 9 | -27.105 | -34.470 | -11.724 | -9.320 | 4.810 | 18.200 | 0.821 | -1.000 | 19 | 0.174 | 1.591 | 1 |
| 10 | -27.105 | -34.470 | -11.724 | -9.320 | 4.810 | 18.200 | 0.820 | -1.000 | 19 | 0.174 | 1.591 | 1 |
| 11 | -26.932 | -33.398 | -9.896 | -9.136 | 1.898 | 18.200 | 0.692 | -1.000 | 18 | 0.050 | 0.357 | 1 |
| 12 | -26.374 | -32.093 | -11.116 | -9.247 | 2.482 | 18.200 | 2.173 | -1.000 | 14 | 0.103 | 2.432 | 1 |
| 13 | -26.374 | -32.093 | -11.116 | -9.247 | 2.482 | 18.200 | 2.178 | -1.000 | 14 | 0.103 | 2.432 | 1 |
| 14 | -26.027 | -32.323 | -11.843 | -9.705 | 4.243 | 18.200 | 0.715 | -1.000 | 19 | 0.154 | 1.414 | 1 |
| 15 | -25.828 | -32.093 | -10.673 | -9.376 | 2.713 | 18.200 | 2.142 | -1.000 | 14 | 0.109 | 2.432 | 1 |
| 16 | -25.656 | -31.832 | -11.030 | -9.314 | 2.920 | 18.200 | 2.217 | -1.000 | 14 | 0.121 | 2.499 | 1 |
| 17 | -25.269 | -32.753 | -9.472 | -9.062 | 2.417 | 18.200 | 0.774 | -1.000 | 17 | 0.062 | 0.408 | 1 |
| 18 | -25.180 | -32.257 | -12.177 | -9.206 | 4.860 | 18.200 | 2.309 | -1.000 | 16 | 0.210 | 1.894 | 1 |
| 19 | -25.136 | -36.193 | -4.934 | -8.667 | 1.058 | 18.200 | 6.207 | -1.000 | 15 | 0.020 | 0.233 | 1 |
| 20 | -25.028 | -36.127 | -4.994 | -8.590 | 1.083 | 18.200 | 6.155 | -1.000 | 15 | 0.021 | 0.239 | 1 |
| 21 | -24.949 | -36.035 | -5.107 | -8.583 | 1.175 | 18.200 | 6.188 | -1.000 | 15 | 0.021 | 0.233 | 1 |
| 22 | -24.836 | -31.393 | -10.343 | -9.182 | 2.482 | 18.200 | 2.013 | -1.000 | 13 | 0.103 | 2.432 | 1 |
| 23 | -24.836 | -31.393 | -10.343 | -9.182 | 2.482 | 18.200 | 2.010 | -1.000 | 13 | 0.103 | 2.432 | 1 |
| 24 | -24.666 | -31.393 | -10.173 | -9.182 | 2.482 | 18.200 | 2.012 | -1.000 | 13 | 0.103 | 2.432 | 1 |
| 25 | -24.666 | -31.393 | -10.173 | -9.182 | 2.482 | 18.200 | 2.007 | -1.000 | 13 | 0.103 | 2.432 | 1 |
| 26 | -24.462 | -32.257 | -11.944 | -9.259 | 5.397 | 18.200 | 2.333 | -1.000 | 16 | 0.240 | 1.894 | 1 |
| 27 | -24.379 | -28.723 | -10.438 | -9.887 | 1.068 | 18.200 | 0.784 | -1.000 | 17 | 0.042 | 0.743 | 1 |
| 28 | -24.110 | -31.788 | -9.435 | -9.119 | 2.631 | 18.200 | 0.894 | -1.000 | 16 | 0.075 | 0.833 | 1 |
| 29 | -24.097 | -31.488 | -8.155 | -8.762 | 0.709 | 18.200 | 1.267 | -1.000 | 19 | 0.010 | 0.155 | 1 |
| 30 | -24.059 | -32.062 | -11.949 | -9.376 | 5.727 | 18.200 | 2.277 | -1.000 | 16 | 0.264 | 1.894 | 1 |

**Table S3:** Scoring values for docking of *Z*-configured azobenzene mannobioside **2** into the closed-gate structure of FimH.

| No. | Total Score | Match-Score | Lipo-Score | Ambig-Score | Clash-Score | Rot-Score | RMS-Value | Simil. Index | #Match | Avg. Volume | Max. Volume | Frag. No. |
|---|---|---|---|---|---|---|---|---|---|---|---|---|
| 1 | -21.629 | -32.441 | -6.000 | -8.104 | 1.316 | 18.200 | 0.000 | -1.000 | 16 | 0.023 | 0.368 | 1 |
| 2 | -21.028 | -31.583 | -6.370 | -8.124 | 1.449 | 18.200 | 0.562 | -1.000 | 14 | 0.027 | 0.373 | 1 |
| 3 | -20.667 | -29.688 | -8.669 | -7.905 | 1.996 | 18.200 | 1.280 | -1.000 | 15 | 0.069 | 1.365 | 1 |
| 4 | -20.367 | -29.176 | -8.595 | -8.544 | 2.347 | 18.200 | 1.314 | -1.000 | 15 | 0.091 | 1.397 | 1 |
| 5 | -20.343 | -27.224 | -11.410 | -7.122 | 1.813 | 18.200 | 3.973 | -1.000 | 9 | 0.036 | 0.505 | 1 |
| 6 | -20.294 | -30.810 | -8.664 | -7.539 | 3.119 | 18.200 | 2.711 | -1.000 | 15 | 0.103 | 1.239 | 1 |
| 7 | -19.913 | -28.876 | -9.322 | -6.770 | 1.454 | 18.200 | 4.235 | -1.000 | 12 | 0.029 | 0.272 | 1 |
| 8 | -19.572 | -31.351 | -5.484 | -7.834 | 1.497 | 18.200 | 1.277 | -1.000 | 13 | 0.029 | 0.433 | 1 |
| 9 | -19.433 | -31.283 | -5.756 | -7.819 | 1.824 | 18.200 | 1.265 | -1.000 | 13 | 0.036 | 0.436 | 1 |
| 10 | -19.289 | -31.357 | -6.031 | -8.700 | 3.198 | 18.200 | 0.913 | -1.000 | 13 | 0.124 | 1.623 | 1 |
| 11 | -19.261 | -30.218 | -8.659 | -7.725 | 3.742 | 18.200 | 2.711 | -1.000 | 14 | 0.144 | 1.726 | 1 |
| 12 | -19.256 | -28.248 | -9.447 | -6.788 | 1.627 | 18.200 | 4.389 | -1.000 | 11 | 0.034 | 0.392 | 1 |
| 13 | -19.074 | -29.401 | -8.115 | -7.754 | 2.596 | 18.200 | 1.463 | -1.000 | 15 | 0.085 | 1.293 | 1 |
| 14 | -19.073 | -30.200 | -7.072 | -6.499 | 1.098 | 18.200 | 2.802 | -1.000 | 15 | 0.017 | 0.213 | 1 |
| 15 | -19.073 | -30.200 | -7.072 | -6.499 | 1.098 | 18.200 | 2.726 | -1.000 | 15 | 0.017 | 0.213 | 1 |
| 16 | -18.928 | -28.939 | -8.841 | -6.950 | 2.203 | 18.200 | 3.917 | -1.000 | 12 | 0.070 | 0.948 | 1 |
| 17 | -18.657 | -29.088 | -7.827 | -7.390 | 2.048 | 18.200 | 3.044 | -1.000 | 13 | 0.050 | 0.699 | 1 |
| 18 | -18.636 | -29.088 | -7.806 | -7.390 | 2.048 | 18.200 | 3.025 | -1.000 | 13 | 0.050 | 0.699 | 1 |
| 19 | -18.544 | -29.238 | -6.309 | -7.606 | 1.010 | 18.200 | 2.447 | -1.000 | 13 | 0.018 | 0.163 | 1 |
| 20 | -18.544 | -29.238 | -6.309 | -7.606 | 1.010 | 18.200 | 2.536 | -1.000 | 13 | 0.018 | 0.163 | 1 |
| 21 | -18.532 | -29.157 | -10.540 | -6.618 | 4.184 | 18.200 | 2.877 | -1.000 | 11 | 0.190 | 2.247 | 1 |
| 22 | -18.369 | -27.137 | -9.210 | -7.423 | 1.801 | 18.200 | 2.706 | -1.000 | 10 | 0.040 | 0.506 | 1 |
| 23 | -18.219 | -28.909 | -7.796 | -7.162 | 2.048 | 18.200 | 2.076 | -1.000 | 12 | 0.048 | 0.699 | 1 |
| 24 | -18.171 | -28.909 | -7.796 | -7.113 | 2.048 | 18.200 | 2.070 | -1.000 | 12 | 0.048 | 0.699 | 1 |
| 25 | -18.063 | -29.804 | -6.325 | -6.930 | 1.396 | 18.200 | 2.757 | -1.000 | 13 | 0.025 | 0.214 | 1 |
| 26 | -18.046 | -29.467 | -6.656 | -6.676 | 1.153 | 18.200 | 3.441 | -1.000 | 13 | 0.018 | 0.197 | 1 |
| 27 | -18.046 | -29.467 | -6.656 | -6.676 | 1.153 | 18.200 | 3.375 | -1.000 | 13 | 0.018 | 0.197 | 1 |
| 28 | -18.012 | -30.467 | -6.022 | -7.753 | 2.630 | 18.200 | 1.721 | -1.000 | 15 | 0.117 | 2.095 | 1 |
| 29 | -17.962 | -28.449 | -8.869 | -6.443 | 2.198 | 18.200 | 4.259 | -1.000 | 10 | 0.052 | 0.496 | 1 |
| 30 | -17.885 | -28.909 | -7.796 | -6.828 | 2.048 | 18.200 | 2.666 | -1.000 | 12 | 0.048 | 0.699 | 1 |

**Table S4:** Scoring values for docking of *Z*-configured azobenzene mannobioside **2** into the open-gate structure of FimH.

| No. | Total Score | Match- Score | Lipo- Score | Ambig- Score | Clash- Score | Rot- Score | RMS- Value | Simil. Index | #Match | Avg. Volume | Max. Volume | Frag. No. |
|---|---|---|---|---|---|---|---|---|---|---|---|---|
| 1 | -28.675 | -36.178 | -11.041 | -10.107 | 5.052 | 18.200 | 0.000 | -1.000 | 18 | 0.183 | 1.604 | 1 |
| 2 | -28.101 | -35.957 | -11.911 | -9.699 | 5.866 | 18.200 | 0.607 | -1.000 | 18 | 0.278 | 1.895 | 1 |
| 3 | -27.461 | -34.986 | -8.537 | -9.270 | 1.733 | 18.200 | 1.984 | -1.000 | 18 | 0.026 | 0.176 | 1 |
| 4 | -27.338 | -35.333 | -8.159 | -9.387 | 1.942 | 18.200 | 1.135 | -1.000 | 18 | 0.047 | 0.658 | 1 |
| 5 | -27.337 | -36.098 | -8.771 | -8.701 | 2.633 | 18.200 | 1.827 | -1.000 | 20 | 0.066 | 0.866 | 1 |
| 6 | -27.336 | -35.422 | -8.846 | -8.652 | 1.984 | 18.200 | 1.153 | -1.000 | 20 | 0.035 | 0.296 | 1 |
| 7 | -27.311 | -33.089 | -11.891 | -9.269 | 3.338 | 18.200 | 1.173 | -1.000 | 17 | 0.081 | 1.103 | 1 |
| 8 | -26.980 | -35.283 | -8.233 | -9.112 | 2.049 | 18.200 | 1.206 | -1.000 | 17 | 0.044 | 0.567 | 1 |
| 9 | -26.805 | -32.947 | -13.663 | -9.641 | 5.846 | 18.200 | 1.635 | -1.000 | 15 | 0.208 | 1.668 | 1 |
| 10 | -26.779 | -34.867 | -8.583 | -8.989 | 2.060 | 18.200 | 1.235 | -1.000 | 18 | 0.035 | 0.245 | 1 |
| 11 | -26.656 | -33.244 | -15.330 | -9.986 | 8.304 | 18.200 | 1.338 | -1.000 | 17 | 0.356 | 2.058 | 1 |
| 12 | -26.549 | -34.269 | -8.961 | -9.240 | 2.320 | 18.200 | 1.353 | -1.000 | 17 | 0.054 | 0.507 | 1 |
| 13 | -26.548 | -34.518 | -8.751 | -8.639 | 1.760 | 18.200 | 1.811 | -1.000 | 17 | 0.034 | 0.440 | 1 |
| 14 | -26.414 | -34.527 | -8.636 | -8.612 | 1.762 | 18.200 | 1.871 | -1.000 | 17 | 0.034 | 0.434 | 1 |
| 15 | -26.242 | -34.448 | -8.513 | -8.706 | 1.825 | 18.200 | 1.241 | -1.000 | 17 | 0.031 | 0.238 | 1 |
| 16 | -26.226 | -35.056 | -8.329 | -8.324 | 1.884 | 18.200 | 3.890 | -1.000 | 17 | 0.033 | 0.275 | 1 |
| 17 | -26.110 | -32.411 | -11.442 | -8.774 | 2.917 | 18.200 | 1.181 | -1.000 | 17 | 0.062 | 0.811 | 1 |
| 18 | -26.072 | -32.609 | -14.988 | -10.020 | 7.946 | 18.200 | 1.378 | -1.000 | 16 | 0.304 | 1.790 | 1 |
| 19 | -25.929 | -34.907 | -7.597 | -9.245 | 2.220 | 18.200 | 1.221 | -1.000 | 18 | 0.052 | 0.746 | 1 |
| 20 | -25.737 | -32.637 | -10.894 | -9.386 | 3.580 | 18.200 | 1.833 | -1.000 | 16 | 0.086 | 0.998 | 1 |
| 21 | -25.682 | -33.273 | -8.441 | -8.614 | 1.047 | 18.200 | 4.184 | -1.000 | 18 | 0.022 | 0.502 | 1 |
| 22 | -25.442 | -34.053 | -8.308 | -8.537 | 1.857 | 18.200 | 2.812 | -1.000 | 16 | 0.038 | 0.535 | 1 |
| 23 | -25.332 | -30.941 | -12.878 | -10.138 | 5.025 | 18.200 | 1.361 | -1.000 | 15 | 0.188 | 1.812 | 1 |
| 24 | -25.318 | -31.356 | -10.634 | -8.341 | 1.414 | 18.200 | 1.356 | -1.000 | 19 | 0.019 | 0.134 | 1 |
| 25 | -25.303 | -33.041 | -8.315 | -8.586 | 1.039 | 18.200 | 2.651 | -1.000 | 17 | 0.021 | 0.455 | 1 |
| 26 | -25.300 | -33.035 | -8.317 | -8.586 | 1.038 | 18.200 | 2.671 | -1.000 | 17 | 0.021 | 0.455 | 1 |
| 27 | -25.285 | -32.073 | -9.889 | -9.078 | 2.155 | 18.200 | 2.017 | -1.000 | 13 | 0.070 | 1.488 | 1 |
| 28 | -25.264 | -33.053 | -8.295 | -8.647 | 1.131 | 18.200 | 4.224 | -1.000 | 17 | 0.022 | 0.485 | 1 |
| 29 | -25.254 | -32.748 | -10.455 | -9.964 | 4.313 | 18.200 | 1.356 | -1.000 | 14 | 0.207 | 1.668 | 1 |
| 30 | -25.249 | -34.097 | -10.945 | -8.911 | 5.104 | 18.200 | 1.892 | -1.000 | 16 | 0.160 | 1.519 | 1 |

## 4. References

[1] a) Reisner, A.; Haagensen, J. A. J.; Schembri, M. A.; Zechner, E. L.; Molin, S., *Mol. Microbiol.*, **2003**, *48*, 933-946; b) The GFP-tagged strain pPKL1162 was constructed in the Klemm group by introduction of the plasmid pPKL174 into strain SAR18; pPKL174 contains the fim gene cluster, which is required for type 1 fimbriae assembly and expression. The chromosome of strain SAR18 from the Reisner group contains the GFP gene, controlled by a constitutive promoter.

[2] Hartmann, M.; Horst, A. K.; Klemm, P.; Lindhorst, T. K., *Chem. Commun.* **2010,** *46*, 330-332.

[3] Tripos, Inc., SYBYL 6.9, 1699 South Hanley Road, St. Louis, MO 63144-2319.

[4] Grabosch, C.; Hartmann, M.; Schmidt-Lassen, J.; Lindhorst, T. K. *ChemBioChem* **2011**, *12*, 1066-1074.

[5] a) Hung, C. S.; Bouckaert, J.; Hung, D.; Pinkner, J., Widberg, C.; Defusco, A.; Auguste, C. G.; Strouse, R.; Langermann, S.; Waksman, G.; Hultgren, S. J. *Mol. Microbiol.* **2002**, *44*, 903-915.; b) Bouckaert, J.; Berglund, J.; Schembri, M.; De Genst, E.; Cools, L.; Wuhrer, M.; Hung, C. S.; Pinkner, J.; Slättegård, R.; Zavialov, A.; Choudhury, D.; Langermann, S.; Hultgren, S. J.; Wyns, L.; Klemm, P.; Oscarson, S.; Knight, S. D.; De Greve, H. *Mol. Microbiol.* **2005**, *55*, 441-455.
